# Determination of Senegenin and Tenuifolin in Mouse Blood by Ultra-High Performance Liquid Chromatography-Tandem Mass Spectrometry and Their Pharmacokinetics

**DOI:** 10.1155/2022/3401355

**Published:** 2022-04-07

**Authors:** Xiuwei Shen, Xiangyi Dai, Yifan He, Congcong Wen, Qingwei Zhang

**Affiliations:** ^1^Ruian People's Hospital, The Third Affiliated Hospital of Wenzhou Medical University, Wenzhou, China; ^2^Laboratory Animal Center, Wenzhou Medical University, Wenzhou, China; ^3^Shanghai Institute of Pharmaceutical Industry Co., Ltd., Shanghai, China

## Abstract

An ultra-high performance liquid chromatography-tandem mass spectrometry (UPLC-MS/MS) method for the determination of senegenin and tenuifolin in mouse blood was developed. The pharmacokinetics of senegenin and tenuifolin in mice after intravenous (5 mg/kg) and oral (60 mg/kg) administration were studied, and the absolute bioavailability was calculated. A CORTECS T3 column was used, with a column temperature set at 40°C. The mobile phase was acetonitrile and 0.1% formic acid. Gradient elution was adopted, using a flow rate of 0.4 mL/min and an elution time of 4 min. Quantitative analysis was performed using electrospray ionization (ESI) with multiple reaction monitoring (MRM) in negative ion mode. Institute of Cancer Research (ICR) mice were bled from the tail vein after intravenous or oral administration of senegenin and tenuifolin. A UPLC-MS/MS method was established to determine the blood concentrations of each drug in mice, and the noncompartmental model was used to fit the pharmacokinetic parameters. Senegenin and tenuifolin showed a good linear relationship (*r* > 0.995) within a concentration range of 5–400 ng/mL in mouse blood. The intraday precision was <12%, the interday precision was <14%, and the accuracy was 87–109%. The recovery was >88%, and the matrix effect was 87–94%. The oral bioavailability of senegenin and tenuifolin in mice was 8.7% and 4.0%, respectively. The established UPLC-MS/MS method is suitable for pharmacokinetic studies of senegenin and tenuifolin in mice.

## 1. Introduction

Senegenin, an active ingredient from the root extract of the Chinese medicine Radix Polygalae, has neuroprotective and neuroreparative effects and is used to treat insomnia, neurasthenia, and dementia [1, 2]. Recently, it has been confirmed that senegenin can alleviate postoperative cognitive dysfunction-related symptoms caused by liver ischemia-reperfusion in rats by increasing the expression of NR2B in the rat hippocampus [3–5]. Tenuifolin is a secondary metabolite of tenuigenin [6]. It has significantly lower hemolytic toxicity than tenuigenin and a strong cholinesterase inhibitory effect, which can inhibit the secretion of *β*-amyloid; however, its water solubility (0.036 mg/mL) and intestinal absorption are poor, and its bioavailability is low, which greatly limit its clinical applications.

Since the concentration of biological samples used to study the pharmacokinetics of traditional Chinese medicines (TCMs) is often extremely low, high performance liquid chromatography and highly sensitive mass spectrometry are used. Triple quadrupole mass spectrometry can significantly improve the detection sensitivity to quantitatively analyze TCM pharmacokinetics [7–9].

Some literature have reported the determination of senegenin or tenuifolin in raw materials or TCM using high performance liquid chromatography (HPLC) [10–13] and biological samples by liquid chromatography-tandem mass spectrometry (LC-MS/MS) [14–16], respectively; however, the simultaneous detection of senegenin and tenuifolin has not been reported. Compared with traditional HPLC analysis methods, blood sample analysis can be completed in only 4 min by UPLC-MS/MS with a high sensitivity and requiring only a small sample. In this study, a UPLC-MS/MS method was established to study the pharmacokinetics of senegenin and tenuifolin in mice.

## 2. Materials and Methods

### 2.1. Reagents and Animals

**Figure 1:**
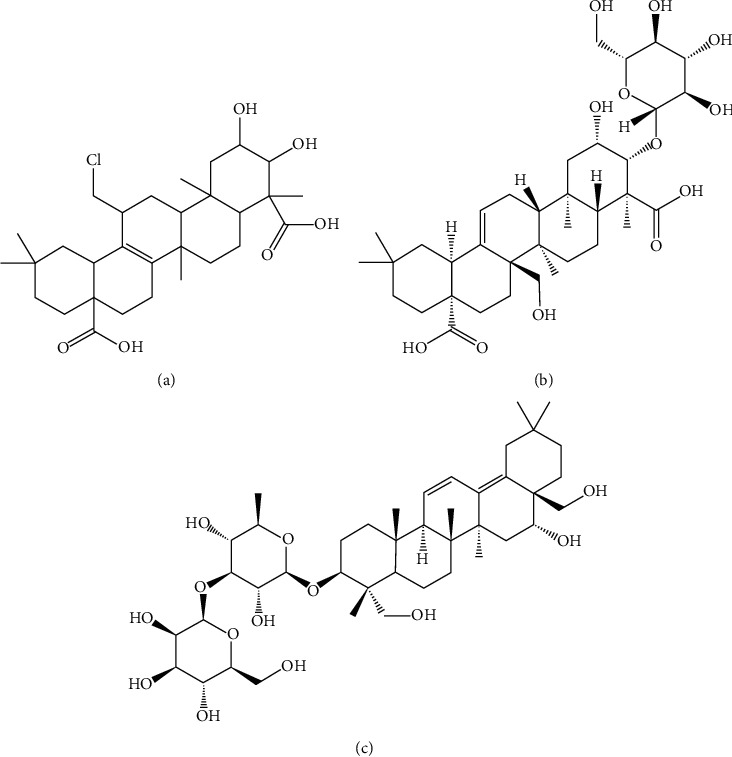
Chemical structure of senegenin (a), tenuifolin (b), and saikosaponin B2 (internal standard) (c).

Senegenin (purity >98%, Figure 1(a)), tenuifolin (purity >98%, Figure 1(b)), and saikosaponin B2 (internal standard, purity ≥98%, Figure 1(c)) were purchased from Chengdu Munster Pharmaceutical Co., Ltd. (Chengdu, China). Chromatographically pure acetonitrile and methanol were purchased from Merck Ltd. (Darmstadt, Germany). Ultrapure water was prepared by a Millipore Milli-Q purification system (Bedford, MA, USA). Institute of Cancer Research (ICR) mice (weight 20–22 g) were purchased from the Animal Experiment Center of Wenzhou Medical University (Wenzhou, China).

### 2.2. UPLC-MS/MS Condition

Acquity I-Class ultra-performance liquid chromatography and XEVO TQS-micro triple quadrupole mass spectrometer (Waters Corp, Milford, MA, USA) were used. Masslynx 4.1 software was used for data acquisition and instrument control.

A CORTECS T3 column (2.1 mm × 50 mm, 1.6 *μ*m) was used, and its temperature was set to 40°C. The mobile phase consisted of acetonitrile and 0.1% formic acid, with a gradient elution program: 0–0.2 min, acetonitrile 10%; 0.2–1.0 min, acetonitrile 10–70%; 1.0–2.5 min, acetonitrile 70–90%; 2.5–2.8 min, acetonitrile 90–10%; 2.8–4.0 min, and acetonitrile 10%. The flow rate was set to 0.4 mL/min, and the elution time was 4 min.

Nitrogen was used as the desolvation gas (900 L/h) and cone gas (50 L/h). The capillary voltage was set to 2.0 kV, the ion source temperature was 150°C, and the desolvation temperature was 500°C. ESI-MS in negative mode MRM was used for quantitative analysis: senegenin *m*/*z* 679.4 > 455.4 (cone voltage 40 V, collision voltage 26 V), tenuifolin *m*/*z* 535.3 > 481.3 (cone voltage 36 V, collision voltage 30 V), and internal standard ion *m*/*z* 825.4 > 617.5 (cone voltage 20 V, collision voltage 40 V).

### 2.3. Preparation of Reference Solution

Stock solutions of senegenin (1.0 mg/mL), tenuifolin (1.0 mg/mL), and saikosaponin B2 (0.1 mg/mL) were prepared in methanol-water (50 : 50). A series of working solutions were prepared by diluting the stock solutions of senegenin and tenuifolin with methanol. The saikosaponin B2 stock solution was diluted with 5% trichloroacetic acid-methanol solution to prepare a solution containing the internal standard saikosaponin B2 with a concentration of 100 ng/mL. All solutions were stored at 4°C.

### 2.4. Preparation of Standard Curve

Appropriate working solutions of senegenin and tenuifolin was added into blank mouse blood to prepare the standard curves of senegenin and tenuifolin (5, 20, 100, 500, 1000, 2000, and 4000 ng/mL). The standard curve concentration range was 5–4000 ng/mL. In the same manner as the standard curve, quality control (QC) samples at three blood concentrations of 10 ng/mL, 450 ng/mL, and 3500 ng/mL were prepared.

### 2.5. Sample Processing

Blood sample (20 *μ*L) was added to a 1.5 mL Eppendorf tube, and 100 *μ*L of 5% trichloroacetic acid-methanol solution (containing 100 ng/mL of the internal standard saikosaponin B2) was added, vortexed for 1.0 min at 4°C, and then centrifuged at 13,000 r/min for 10 min. The supernatant (80 *μ*L) was taken into the liner tube of the injection bottle, and 3 *μ*L was injected into the UPLC-MS/MS system.

### 2.6. Pharmacokinetics

Twelve mice were randomly divided into two groups, with six mice in each group: one group was administered intravenously (5 mg/kg) and the other group was administered orally (60 mg/kg). After 0.0833 h, 0.5 h, 1 h, 2 h, 3 h, 4 h, 6 h, 8 h, and 12 h, 20 *μ*L of blood was collected from the tail vein of mice after intravenous or oral administration of senegenin and tenuifolin, respectively. The samples were stored frozen at −20°C in 1.5 mL Eppendorf tubes. The blood concentration data of mice in each group at different times were substituted into Drug and Statistics (DAS) 2.0 software (China Pharmaceutical University), and the pharmacokinetic parameters were fitted using a noncompartmental model.

## 3. Results

### 3.1. Selectivity

**Figure 2:**
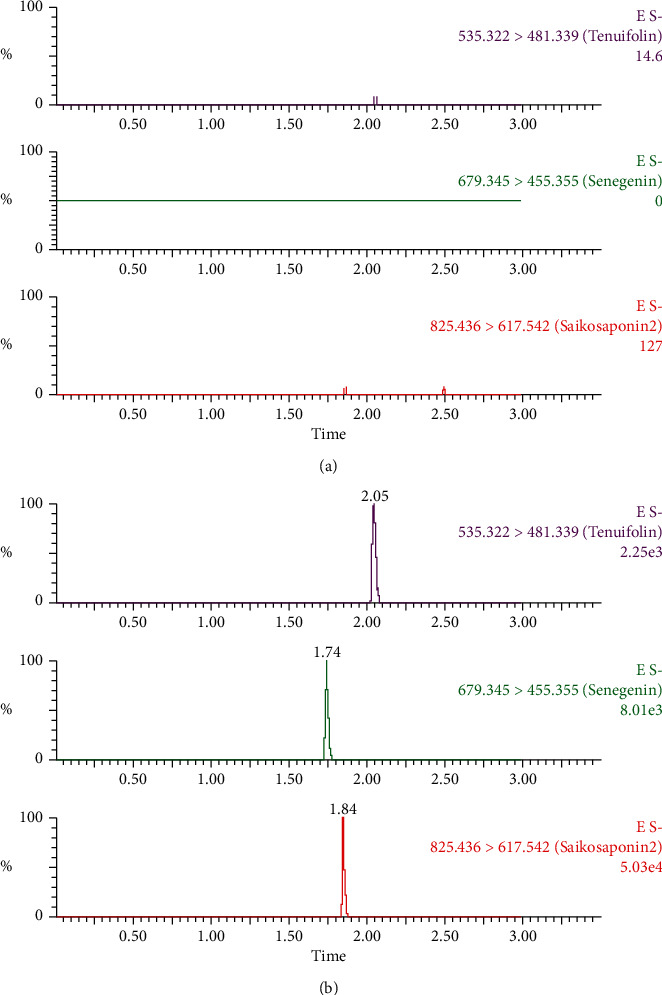
Ultra-high performance liquid chromatography-mass spectrometry spectra of senegenin, tenuifolin, and saikosaponin B2 (internal standard) in mouse blood. (a) Blank blood. (b) Blank blood spiked with senegenin, tenuifolin, and saikosaponin B2 (internal standard).

Figure 2 shows that the retention times of senegenin, tenuifolin, and internal standard were 1.74 min, 2.05 min, and 1.84 min, respectively. No obvious impurities or endogenous substances interfered with the detection, indicating that the method has good selectivity.

### 3.2. Linear

A series of standard solutions of different concentrations were prepared as working solutions, and the standard concentration range was 5–4000 ng/mL. Under the same conditions as the blood samples, each peak area was measured, and the ratio of the peak area of the sample and internal standard was used to draw a standard curve. The linearity of the data was evaluated by the standard curve. The standard curve equation of senegenin in mouse blood was *y* = 0.00025*x* + 0.00015 and *r* = 0.9985. The standard curve equation of tenuifolin was *y* = 0.000068*x* + 0.000053 and *r* = 0.9964. Among them, *y* represents the peak area ratio of senegenin and tenuifolin to the internal standard, and *x* represents the concentration of senegenin or tenuifolin in blood. The lower limit of quantification of senegenin and tenuifolin in mouse blood was 5 ng/mL.

### 3.3. Precision, Accuracy, Recovery, and Matrix Effects

The precision and accuracy were assessed by six replicate measurements of blood samples for these three QC sample concentration levels. Precision was expressed as the relative standard deviation (RSD), and the intraday and interday precisions were determined by measuring QC samples for three consecutive days. The intraday and interday accuracies of the QC samples were determined by measuring how well the mean of the QC samples matched the true value over three consecutive days. In mouse blood, intraday precision was <12%, interday precision was <14%, accuracy was 87–109%, recovery was >88%, and the matrix effect was 87–94% (Table 1).

### 3.4. Stability

The accuracy of detecting senegenin and tenuifolin in mouse blood at room temperature for 2 h, −20°C for 30 d, and freeze-thaw stability tests was within the range of 88–109%, and the RSD was within 12% (Table 2). This indicates that senegenin and tenuifolin have good stability in mouse blood samples.

### 3.5. Pharmacokinetics

**Figure 3:**
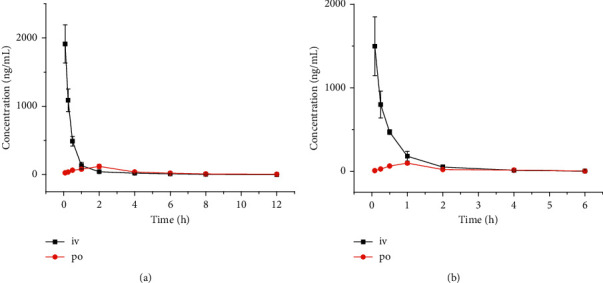
The blood concentration-time curves after intravenous (iv, 5 mg/kg) and oral (po, 60 mg/kg) administration of senegenin (a) and tenuifolin (b) in mice.

Figure 3 shows the drug concentration-time curves obtained from the pharmacokinetic data of senegenin and tenuifolin after intravenous or oral administration in mice. Noncompartmental models were used to fit the main pharmacokinetic parameters (Table 3). The half-lives of senegenin and tenuifolin in mice were relatively short, indicating they were rapidly metabolized. The oral bioavailability of senegenin and tenuifolin in mice was 8.7% and 4.0%, respectively, indicating a low oral bioavailability.

## 4. Discussion

The application of LC-MS/MS technology to pharmacokinetics research is a very important step in the history of pharmacokinetics development [17]. The sensitivity and specificity of effective substance detection in biological samples have been greatly improved. In the past, due to insufficient economic, scientific, and technological conditions, HPLC was typically used for detection, but its sensitivity and specificity were insufficient. There are many active ingredients that cannot be detected at a certain concentration, so it is difficult to accurately express dynamic changes in the absorption, distribution, metabolism, and excretion of active pharmaceutical ingredients in vivo. LC-MS/MS is characterized by a high detection sensitivity, and the samples can display a good response signal in LC-MS/MS, even at low concentrations [18–20]. The specified precursor ion and product ion transition pair can be monitored, resulting in high specificity [21, 22].

ESI negative ion and positive ion modes are often used in methodological research. Comparing the positive and negative modes, ESI negative ion has higher sensitivity for detecting senegenin and tenuifolin. When using optimized liquid chromatography conditions, the column and mobile phase are decisive. In this experiment, we tried different chromatographic columns such as BEH C18 and CORTECS T3 with different mobile phase compositions and found that CORTECS T3 (2.1 mm × 50 mm, 1.6 *μ*m) and a mobile phase of acetonitrile-0.1% formic acid had appropriate peaks.

Different precipitation solvents, methanol, acetonitrile, methanol-acetonitrile (1 : 1), methanol-acetonitrile (1 : 9), 5% trichloroacetic acid in methanol, and 5% trichloroacetic acid in water were screened during sample processing. The recovery of 5% trichloroacetic acid-methanol was the highest, so it was selected as the protein precipitant.

Lin et al. developed a rapid and specific LC-MS/MS method for the simultaneous determination of polylactic acid, senegenin, and 3,6′-dinaphthylsucrose (DSS) in rat plasma, which took 17 min per sample [14]. This method was applied to the pharmacokinetic studies of polygallic acid, senegenin, and DSS. The half-life *t*_1/2_ of senegenin was 12.66 ± 5.29 h, and the mean *C*_max_ was 89.35 ± 36.3 ng/mL. These were different from half-life *t*_1/2_ of senegenin 2.6 ± 0.6 h in this work, which may be affected by mixing with different compounds and the different pharmacokinetics of mice and rats.

Ma et al. established and validated a sensitive, reliable, and accurate LC-MS/MS method in negative ion mode for the quantification of tenuifolin in rat plasma and tissue, and each sample detection took 6 min [15]. The method was successfully applied to study the pharmacokinetics and tissue distribution of pure tenuifolin in rats. Pharmacokinetic studies showed that the absolute bioavailability was low (0.83 ± 0.28%) when the systemic malabsorption rats were administered orally to observe circulation. Wang et al. developed a sensitive LC-MS/MS method for the simultaneous determination of the three main active components of *Polygala* saponin hydrolysates in rats, 3,4,5-trimethoxycinnamylic acid (TMCA), *p*-methoxycinnamylic acid (PMCA), and tenuifolin (TF), which required 6 min per sample analysis [16]. Pharmacokinetic studies have shown that TMCA, PMCA, and tenuifolin can be rapidly absorbed into blood, reaching peak concentrations at around 9.1, 9.0, and 24.0 min, respectively. The oral bioavailability of tenuifolin (2.0%) was lower than that of TMCA (90.1%) and PMCA (96.5%), but its residence time in the body (*t*_1/2_, 4.8 h, oral dose) was longer than that of TMCA (0.6 h) and PMCA (0.9 h). In this study, the *t*_1/2_ of tenuifolin was 1.1 ± 0.2 h, and its bioavailability was 4.0%, which were different from the previous report.

In this experiment, a UPLC-MS/MS method was established, in which multiple reaction monitoring and ESI negative ion mode were used to determine the plasma concentrations of senegenin and tenuifolin in mice. The noncompartmental model was used to fit the pharmacokinetic parameters, and the bioavailability was calculated. The analysis showed that senegenin and tenuifolin were rapidly metabolized in mice, and their oral bioavailability was low.

## Figures and Tables

**Table 1:** Accuracy, precision, matrix effect, and recovery of senegenin and tenuifolin in mouse blood.

Compound	Concentration (ng/mL)	Accuracy (%)		Precision (RSD%)		Matrix effect (%)	Recovery (%)
		Intraday	Interday	Intraday	Interday		
Senegenin	5	108.8	91.3	11.1	11.5	87.7	94.2
	10	104.6	102.0	10.8	6.6	87.3	94.9
	450	102.8	96.4	6.7	11.3	89.0	90.1
	3500	96.1	103.7	6.7	10.9	93.4	93.1
Tenuifolin	5	109.5	87.0	11.7	13.8	88.4	97.7
	10	96.7	107.4	5.4	12.4	93.4	95.2
	450	108.0	89.1	9.8	9.0	86.1	88.9
	3500	93.5	106.3	10.5	12.0	88.4	96.4

**Table 2:** Stability of senegenin and tenuifolin in mouse blood.

Compound	Concentration (ng/mL)	Autosampler (4°C, 12 h)		Ambient (2 h)		−20°C (30 d)		Freeze-thaw	
		Accuracy	RSD	Accuracy	RSD	Accuracy	RSD	Accuracy	RSD
Senegenin	10	98.0	3.6	98.5	3.4	93.1	10.3	88.7	11.9
	450	100.6	7.3	107.7	8.3	106.6	10.0	106.2	11.5
	3500	102.2	3.0	97.9	1.5	103.8	8.0	99.3	8.1
Tenuifolin	10	101.8	3.3	99.5	4.1	108.3	11.3	90.0	11.4
	450	102.6	6.8	103.8	2.6	94.6	3.7	104.7	4.4
	3500	97.8	2.5	98.5	5.0	104.1	10.4	90.3	10.4

**Table 3:** The main pharmacokinetic parameters of senegenin and tenuifolin after intravenous (iv) and oral (po) administration in mice.

Compound	Group	AUC_(0-t)_ (ng/mL^*∗*^h)	AUC_(0-∞)_ (ng/mL^*∗*^h)	*t*_1/2z_ (h)	*CL*_z/F_ (L/h/kg)	*V*_z/F_ (L/kg)	*C*_max_ (ng/mL)
Senegenin	PO, 60 mg/kg	434.2 ± 67.8	454.7 ± 66.0	2.6 ± 0.6	134.3 ± 21.8	503.0 ± 152.0	119.7 ± 30.8
	IV, 5 mg/kg	994.3 ± 116.0	998.0 ± 116.5	1.6 ± 0.4	5.1 ± 0.7	12.2 ± 4.1	1910.6 ± 279.2
Tenuifolin	PO, 60 mg/kg	170.8 ± 20.8	176.1 ± 21.2	1.1 ± 0.2	343.8 ± 38.7	554.7 ± 23.3	98.3 ± 19.0
	IV, 5 mg/kg	845.4 ± 171.2	849.4 ± 172.6	0.8 ± 0.2	6.1 ± 1.4	7.2 ± 2.2	1497.3 ± 351.2

## Data Availability

The data used to support the findings of this study are included within the article.
